# What attributes should be included in a discrete choice experiment related to health technologies? A systematic literature review

**DOI:** 10.1371/journal.pone.0219905

**Published:** 2019-07-18

**Authors:** Marta Trapero-Bertran, Beatriz Rodríguez-Martín, Julio López-Bastida

**Affiliations:** 1 Department of Nursing, Physiotherapy and Occupational Therapy, University of Castilla La-Mancha (UCLM), Talavera de la Reina (Toledo), Spain; 2 Research Institute for Evaluation and Public Policies (IRAPP), Universitat Internacional de Catalunya (UIC), Barcelona, Spain; 3 Faculty of Health Sciences, University College Dublin, Dublin, Ireland; Universita degli Studi di Firenze, ITALY

## Abstract

Discrete choice experiments (DCEs) are a way to assess priority-setting in health care provision. This approach allows for the evaluation of individuals’ preferences as a means of adding criteria to traditional quality-adjusted life year analysis. The aim of this systematic literature review was to identify attributes for designing a DCE in order to then develop and validate a framework that supports decision-making on health technologies. Our systematic literature review replicated the methods and search terms used by de Bekker-Grob et al. 2012 and Clark et al. 2014. The Medline database was searched for articles dated between 2008 and 2015. The search was limited to studies in English that reflected general preferences and were choice-based, published as full-text articles and related to health technologies. This study included 72 papers, 52% of which focused on DCEs on drug treatments. The average number of attributes used in all included DCE studies was 5.74 (SD 1.98). The most frequently used attributes in these DCEs were improvements in health (78%), side effects (57%) and cost of treatment (53%). Other, less frequently used attributes included waiting time for treatment or duration of treatment (25%), severity of disease (7%) and value for money (4%). The attributes identified might inform future DCE surveys designed to study societal preferences regarding health technologies in order to better inform decisions in health technology assessment.

## Introduction

In health care systems around the world, decision-makers are faced with competing demands and insufficient resources, even in the wealthiest countries. In these circumstances, it is not possible to provide all available and potentially beneficial health care to those who could benefit from it, and priority-setting is therefore needed. Policy-makers should take into account the views of the general population in setting health priorities, as is done in the United Kingdom [1]. Public involvement in health care decision-making should be a policy objective, although there is an absence of empirical evidence on how society might value different health interventions [2].

There is substantial literature on the different methods available to engage the public in health care decision-making [3–5]. As noted by Whitty et al. [6], Ryan and colleagues provide a comprehensive systematic review and comparative assessment of the methods that can be used to elicit public preferences for health care [7], concluding that “there is no single, best method to gain public opinion”. Nevertheless, they do make recommendations regarding the appropriateness of selected qualitative and quantitative techniques. Two of their preferred methods, the citizens’ jury and discrete choice experiments (DCEs), have been gaining prominence in the health literature in recent years [6, 8–12]. Each is associated with a number of features that make them particularly attractive for public engagement [6], rendering them worthy of further consideration. The DCE approach was chosen for this systematic literature review because it was aimed at informing a pilot study using a discrete choice experiment to quantify individual preferences regarding the use of public funding for orphan drugs.

The DCE has become a useful instrument for quantifying preferences related to health care priority-setting [6, 8, 13–16]. It has been used to (1) measure preferences regarding a health care service, (2) measure preferences regarding the distribution of health care within a population and (3) to assess preferences for the funding of health care [17–19]. Although use of the method by policy-makers has not yet become widespread, it has been applied to elicit social preferences for health care funded with public money [20].

A DCE survey can be administered relatively easily to a large, randomly selected representative sample of the population [7]. It is arguably a less resource-intensive method of community engagement than many other approaches, although resources and costs would likely be high for large sample sizes. A DCE measures not only the direction of preferences around a topic (e.g., Should health gains attributed to young children be weighted more heavily than those attributed to older people?), but also the relative strength of preferences for one policy choice alternative compared with another (e.g., How much extra weight should be attributed to young children?), as well as the trade-offs that respondents would be willing to make between different characteristics of that choice. The usefulness of most preference-based approaches (including DCEs) may be limited when the respondents represent what might be called a naïve sample of the general public–that is, one comprising individuals who lack personal knowledge or experience on the issue, and thus little weight can be given to the results [7, 21].

A DCE provides a different way–compared with other approaches, such as small‐scale discussions or focus groups–to assess priority-setting based on the valuation of some attributes. DCEs are based on the assumption that health care interventions, services or policies can be described by their attributes and that an individual’s valuation depends upon the levels of these attributes. In a DCE, respondents are asked to choose between two or more alternatives. The resulting choices reveal an underlying utility function. For example, the DCE approach allows for the evaluation of individuals’ preferences for adding criteria to traditional quality-adjusted life year (QALY) analysis. The DCE approach also facilitates greater knowledge of the relative importance of the various attributes and the trade-offs that individuals are willing to make between these attributes.

This type of research and its applications are crucial for identifying the current impact of new health technologies on health and economics and, therefore, for assessing their effectiveness. DCEs also afford an opportunity to assess societal preferences. This type of research could serve as the basis for an integrated and harmonized approach to assessing public policies on new health technologies in the European Union.

De Bekker Grob et al. [8] published a recent review on preferences of consumers, patients and health professionals for all types of health care resources. They focused on the experimental design of DCEs, estimation procedures, the validity of responses and the definition of the attributes and their respective levels that should be used for DCEs on health technologies. The attributes found in their review were monetary measure, time, risk, health status domain, health care, and other. No further description of the attributes was given, so there were no well-defined inputs to be used to design a DCE for a particular context. Clark et al. [22] published a more recent DCE review. This paper updated the paper by de Bekker Grob et al. [8] and explored trends in DCEs used in health economics. It concluded that the use of DCEs in health care continues to grow dramatically across a broad range of countries. Thus, DCE results may be influencing decisions in a wider range of geographical settings. Little description and detail regarding attributes and their respective levels were provided for inclusion in future DCE exercises. There have been several literature reviews of DCEs in health care in general (such as de Bekker Grob et al. and Clark et al.) [8, 22], but not of DCEs in health technology assessment (HTA). Decisions regarding HTA also involve public resources, however, and it is therefore important to establish approaches for prioritizing health technology resources. Accordingly, determining the attributes that should be considered in DCEs to inform HTA decisions should be a current research concern.

## Materials and methods

This systematic literature review was conducted using the search terms and methods used in two recent published systematic reviews on DCEs covering the periods 2001–2008 [8] and 2009–2015 [22]. These methods involved the use of the Medline Ovid database to identify DCE studies on health care or health economics. These studies used the same search terms used by Ryan and Gerard [23], reflecting the different terms applied to refer to DCEs. The search terms included were “discrete choice experiment(s)”, “discrete choice model(l)ing”, “stated preference”, “part-worth utilities”, “functional measurement”, “paired comparisons”, “pairwise choices”, “conjoint analysis”, “conjoint measurement”, “conjoint studies” and “conjoint choice experiment(s)”.

In this study, the same database used in de Bekker Grob et al. [8] and Clark et al. [22] was used to search for articles published from January 2008 to December 2015. The same key words were also used. Papers in English and Spanish were retrieved, although the search terms used were in English only. Any paper explaining a DCE on health technologies was included. Review papers were excluded from the analysis but kept for the discussion section of this paper. De Bekker-Grob et al. [8] and Clark et al. [22] included studies that were choice-based and published as full-text articles and that applied to health care or health economics in general. Our review focused on health technologies and thus had a more limited scope. The search was extensive with respect to health care and health economics in general, but only papers related to health technologies were included in our systematic review. The objective was to evaluate DCEs on health technologies that reflected the preferences of patients, policy-makers, providers and the general public. Papers were excluded if they had the following characteristics: (a) they were duplicates; (b) they were not choice- or preference-based or they merely provided measurements but no descriptions of attributes; (c) neither the full text nor an abstract was found; (d) they did not apply to health technologies or to rural areas of developing countries; and (e) they did not involve human respondents. Grey literature was also searched using Google Scholar, although unfortunately no results were found. Each abstract and paper selected was carefully peer-reviewed, and data extraction was systematically and independently performed by two researchers. Whenever there was a discrepancy, papers were reviewed a second time to reach a consensus. Excel was used to summarize the results of this systematic literature review. A data extraction form included questions on the following: background (e.g., quartile of impact factor); sampling and sample characteristics (e.g., illness of respondents); general design of the DCE (e.g., number of attributes and description of attributes and levels covered); experimental design (e.g., method for creating choice sets); design validity (e.g., estimation procedure, model); and qualitative methods for enhancing the DCE process and results (e.g., pretesting of the DCE questionnaire). However, only the general design and experimental design features were presented in the results and discussion sections. Considerations relating to design validity and qualitative methods used to enhance the DCE process were beyond the scope of this paper.

The specific details of the template were dynamically adjusted during the piloting process, which included the revision of a few papers. Data were extracted in free-text form with no limitations on the number of free-text fields and as little categorization of data as possible to avoid the loss of detailed information. Descriptive analysis was undertaken to describe the most common attributes used and their corresponding levels. The attributes and levels for the DCE questionnaire on HTA are presented in a summary table. The table shows the attributes found in this literature review and the attributes identified in the previously published systematic literature reviews [8, 22].

To assess the methodological quality of the systematic literature review, the PRISMA checklist was used [24]. In addition, a DCE quality assessment tool [25] was used to assess the validity of the studies and their attributes and levels.

This approach was devised following the guidance of Mandeville et al. [25], who covered all four key stages of a DCE (choice task design; experimental design; conduct; and analysis) using a list of 13 criteria drawn from an earlier study [26]. The authors assessed whether each criterion for each study was met. If the criterion was met, it was indicated with a green colour. If there was insufficient information to judge whether a criterion was met, then an orange colour was used. A red colour indicated that the criterion was not met. This type of qualitative analysis is important for validating the results from this systematic literature review.

## Results

**Fig 1:**
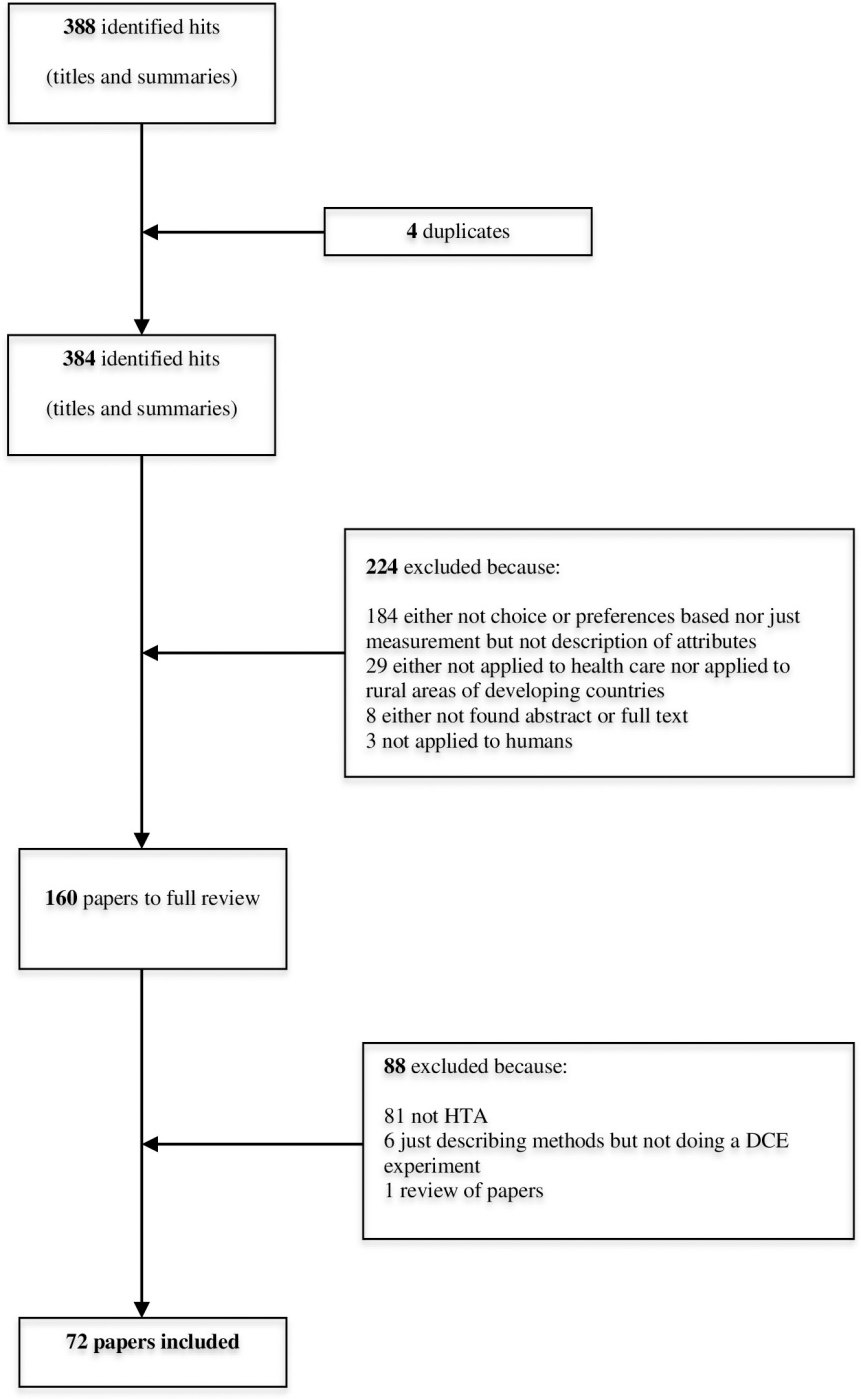
Number of hits identified by the literature review.

Fig 1 shows the flowchart for the identification of studies, with reasons for exclusion.

Overall, the search strategy identified 384 titles (after duplicates were excluded) from a pool of studies with the potential for inclusion in this review. Based on the abstracts, 160 papers were ordered and manually reviewed. Of these 160 articles, 72 were included in this study [14, 27–96]. S1 Table, included in the supplemental material, provides details of the PRISMA checklist used to assess the methodological quality of the systematic literature reviews.

The number of studies published on DCEs on general preferences regarding health technologies over time is as follows: 18 articles (25%) were published before 2010, 30 articles (41.6%) appeared between 2010 and 2011, and the remaining 21 articles (29%) were published between 2012 and 2013. The years with the greatest research output were 2011 and 2012 (16 articles and 15 articles published, respectively). The average sample used across the 72 studies included 299 individuals with a mean age of 59.6 years; an average of 44.88% of the respondents were female. In seven studies, only the age interval was reported; in those cases, the average of the age interval was taken. The largest number of the DCEs identified were conducted in the Netherlands, although significant numbers were also conducted in the United Kingdom, Canada, Germany and the United States. The 72 papers covered 30 different diseases, such as chronic obstructive pulmonary disease (COPD), depression and hepatitis B. The most studied preferences related to cancer (26%), followed by attention deficit disorder (4%) and osteoporosis (4%). Only one paper [61] was found that examined preferences relating to orphan drugs for rare diseases. S2 Table, included in the supplemental material, provides more detailed information about the health technologies, the attributes and the levels for each paper.

**Fig 2:**
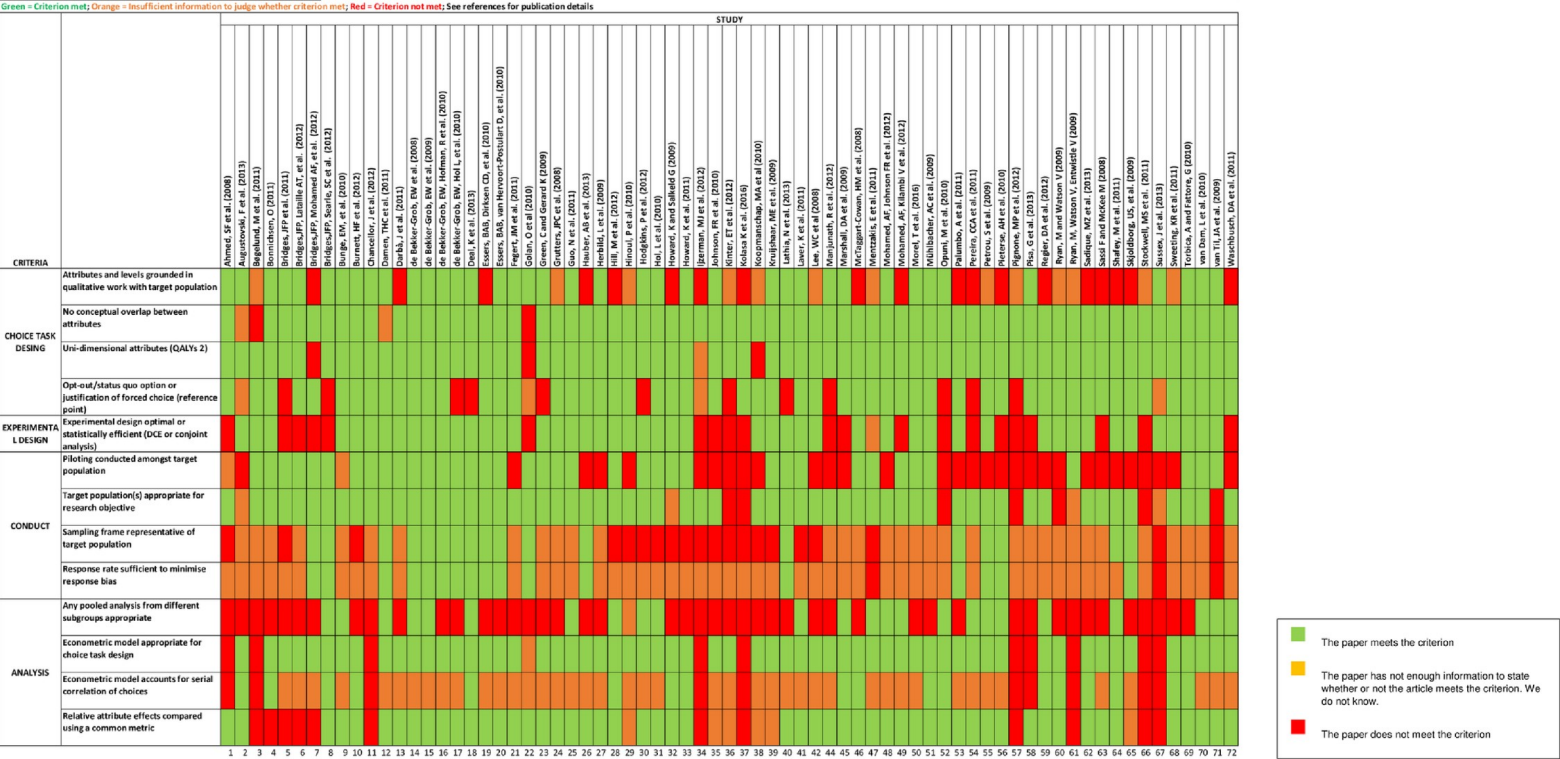
Validity assessment of included studies.

Fig 2 presents the validity assessment for all included studies. Overall, while the choice task design and the experimental design of the studies were more robust than expected, there were significant weaknesses with regard to conduct and analysis of the studies. In terms of choice task, attributes and levels were identified through qualitative work with the target population in 24.8% of the studies. In 16% of the studies, there was no opt-out or status quo option, nor any justification of a forced choice for the attributes selected. In terms of the experimental design, 27% of papers did not have a design that was optimal or statistically efficient. However, most of the relevant problems with the validity of the papers pertained to the pilot testing conducted among the target population and the lack of a pooled analysis from different subgroups: in 42% of the papers the authors did not conduct a pilot test to inform the design of the questionnaire, and 63% did not include any pooled analysis from different population subgroups. In an assessment of the validity of experimental design and analytic approach, it is necessary to examine current practices in DCEs.

Most of the studies (53%) assessed societal preferences regarding pharmacological treatments; the rest focused on medical devices. Of the latter, 17% related to diagnostic technologies and 26% to therapeutic technologies (specifically, 13% were on assistive technology devices used directly by patients, 5% were on medical devices to assist medical professionals, and 4% were on artificial body parts implanted through medical procedures). The average number of attributes was 5.74 (SD 1.98), with a minimum value of 2 and a maximum value of 12. Each attribute had an average of 3.26 levels (SD 1.11), with a minimum value of 1 and a maximum value of 18. The six most common attributes used in the DCEs (n = 72) were (a) improvement in health (78%), (b) side effects (57%), (c) costs (53%), (d) waiting time for treatment or duration of treatment (25%), (e) severity of disease (7%), and (f) value for money (4%). When the focus was only on papers that assessed preferences in relation to drugs (n = 36), the relative importance of the attributes remained the same: improvement in health (55.56%), costs (50%), adverse events (41.67%), and mode of administration (22.22%), followed by discomfort and pain (16.67%), treatment duration (19.44%) and waiting time (2.78%). These attributes reflected the general preferences of several groups, including patients, the general public patients and other stakeholders. The majority of the papers (n = 60) referred to patients’ preferences. In terms of attributes revealing preferences related to efficiency, availability of other treatments and value for money were considered relevant attributes [14]. Availability of other treatments refers to the existence of alternative treatments for the same disease. Value for money refers to how efficiently resources are used (e.g., doctor time, hospital beds, drugs) in the national health system and is based on the relationship between the cost of treatment and the health benefits it provides. Hence, although these two attributes were not the most commonly used, they were also considered for inclusion in the DCE survey.

In terms of levels, the papers reviewed most commonly referred to the following levels of administration: oral, subcutaneous, intravenous or injection. These papers also referred to the following levels of pain or discomfort: none, mild, moderate or severe. Therefore, the terms mild, moderate, and severe were adopted to describe the levels for as many attributes as possible. See Table 1 for details on the most used attributes and their respective levels. This table also includes the attributes and levels described in the two previously published systematic reviews [8, 22]. Both studies highlight the monetary measure and the time- and health care-related attributes as the most frequent ones to be considered in a DCE.

**Table 1:** Attributes and levels for use in a DCE questionnaire on HTA.

Attributes	n	Levels
**From previous systematic literature review on health care (de Bekker-Grob et al. 2012 (DB) N = 114; Clark et al. 2014 (C) N = 179)**		
Monetary measure	80 (DB); 98 (C)	?
Time	83 (DB); 113 (C)	?
Risk	47 (DB); 73 (C)	?
Heath status domain	81 (DB); 56 (C)	?
Health care	107 (DB); 73 (C)	?
Other	20 (DB); 24 (C)	?
**From this systematic literature review on HTA (N = 72)**		
Improvement in health	56	a) Large improvement
		b) Moderate improvement
		c) Small improvement
		d) Very small improvement
Side effects	41	a) Few side effects
		b) Moderate side effects
		c) Many side effects
Cost (price) of treatment	38	a) Zero increase in tax/co-payment
		b) Low increase in tax/co-payment
		c) Moderate increase in tax/co-payment
		d) High increase in tax/co-payment
Waiting time for the treatment or treatment duration	18	a) Short
		b) Moderate
		c) Long
Severity of the disease	5	a) Moderate
		b) Severe
Value for money	3	a) Very good
		b) Fairly good
		c) Fairly poor
		d) Very poor

Only 10 papers (13.8%) offered partial access to the questionnaire used to carry out the DCE and the specific questions formulated for respondents. The rest of the papers did not offer access to the survey. The most common means of administering the survey was a self-report questionnaire (35.29%); questionnaires were administered through an interviewer in 7.35% of cases or a computer in 8.82%. The rest of the papers did not report the mode of survey administration.

Regarding experimental design, 43% (n = 31) of the studies had a fractional factorial design, whereas 13% (n = 10) had a full factorial design. The remaining 27 studies did not report the type of DCE design. Thirty-one studies measured main effects only, whereas seven studies reported main effects with 2-way interactions. In 32% of the DCEs reviewed, the effects evaluated were not reported. More than half of the papers (55%) used orthogonal arrays to create choice sets, while 14% used D-efficiency methods. Five papers combined two methods: either orthogonal arrays with D-efficiency or other methods. Three papers used other methods to create choice sets, and 18 did not explain the method used. In terms of the estimation procedure, the multinomial logit was the most common model used to analyse DCE preferences (22%), followed by random effects logit (13%) and logit (12%).

## Discussion

This study fills a gap in systematic reviews of the literature aimed at identifying the most relevant attributes and levels for measuring public preferences regarding health technologies by means of a DCE. Six attributes were identified from de Bekker Grob et al. (2012) [8] and Clark et al. (2014) [22]: health status domain, monetary measure, time, risk, health care and other, some of which were too subjective to build questions for a DCE survey. No levels were defined or detailed in these papers and no additional information was given concerning the definitions of these attributes. In contrast, our literature review found the following attributes: improvement in health, side effects, cost (price) of treatment, waiting time for treatment or treatment duration, severity of disease and value for money. The attributes included in the previous systematic reviews are too wide and general to understand. No definitions were provided by the authors, so it was difficult to evaluate the complementarity between the results of the two systematic literature reviews, even though they used the same literature review methods. It will be important for future research to describe these attributes and their respective levels in as detailed a manner as possible, so that they can be applied with no uncertainties regarding what is encompassed in their definition. In addition, a complete description will be helpful in providing information for the design of future DCEs on HTA. Because public preferences might change greatly over time, depending on current situations worldwide, it was decided to incorporate papers published between 2008 and 2015 –i.e., a period of 7 years. Regarding the optimum number of attributes to include in a DCE, Marshal et al. [97] identified and described recent applications of conjoint analysis to determine what combination of a limited number of attributes was most influential on respondent choice or decision-making. In their review, they found that most surveys included 6 attributes, with the number ranging from 3 to 16. Therefore, it seems that a larger number of attributes should be used to better capture the criteria on which people base their preferences related to health care. However, many attributes make the decision task more difficult and hence render the outcomes less reliable. The number of attributes found in this systematic review–six–seems a sensible and adequate number to be potentially included in a DCE.

Orphan drugs are unlikely to be efficient (provide value for money) due to the high price paid for often modest effectiveness. It is important to identify all appropriate criteria that will help in the “correct” evaluation of the potential impact and benefit generated in society. Unfortunately, only one paper [61] was found that studied preferences relating to orphan drugs for rare diseases. The authors investigated public preferences regarding public funding for orphan drugs used to treat both rare and common diseases, using a convenience sample of university students. They found that when all other variables were held constant, the respondents did not prefer to have the government spend more for orphan drugs used to treat rare diseases and that they weighted the relevant attributes of coverage decisions similarly for both rare and common diseases. More DCEs on orphan drugs should be conducted to generate more evidence on the particular attributes and levels for this kind of drug.

The inclusion of either cost or improvement in health and value for money as attributes helps to capture the preferences of respondents, although it could lead to double-counting. None of the papers found in this systematic review included either combination of those attributes; however, it is important to be aware of the potential for double-counting that can occur as a result of the inclusion of such similar attributes.

Although there were significant weaknesses in terms of the validity assessment of the included studies, important and essential issues–such as no overlap between the attributes, the use of unidimensional attributes in the questionnaires, the use of the correct target population and the appropriate use of an econometric model for the choice task design–were common characteristics for most of the studies. Hence, despite some weaknesses regarding validity, the most important criteria for these types of studies were included overall.

DCEs have been previously used in other published studies to gain insight into the criteria that were important for decision-makers in health care priority-setting [14, 40, 52, 64, 65]. These five papers were included in this literature review. The rest of the papers (n = 67) focused on patient preferences. The type of attributes used in the papers that focused on policy-makers’ opinions were quite different from those that sought to identify patients’ preferences. For instance, six intervention-related attributes were included in a paper [73] that measured the preferences of policy-makers and other health professionals, including disease severity, budget impact, cost-effectiveness (incremental cost per QALY and number of QALYs gained per patient), uncertainty regarding the probability of doubling costs per QALY, national savings in costs related to absence from work per year and the composition of the health gain. Disease severity and cost of treatment are included in both literature reviews, as is health improvement; however, questions related to cost-effectiveness or national savings when the target audience includes patients are more difficult to ask. For that reason, availability of other treatments and value for money were included as relevant attributes. Attributes might differ, depending on the survey target audience. In this case, all audiences have been included.

Subsequent research is needed to further develop DCE attributes and levels for various specific technologies and diseases. One possible approach might be to investigate in a more in-depth manner the methods that led to the selection and identification of attributes in DCE studies (e.g., focus groups, interviews, literature, expert opinion), which could then be informative for future DCEs. Another approach might be to conduct DCEs for different diseases among different types of audiences to assess and validate attributes and thus help to inform future priority-setting decisions.

## Conclusions

This systematic literature review was performed to identify the attributes that may better help decision-makers and patients to identify the criteria leading to decisions about health technologies. This study revealed that attributes such as improvements in health, treatment side effects, treatment cost (price), waiting time for treatment or treatment duration, severity of disease and value for money can be considered to better capture and describe societal preferences in relation to HTA. This topic is of interest for preference practitioners, as it can help them, first, to build the best survey on health technology and, then, to aid public decision-makers in identifying the treatments that should be implemented or funded, in accordance with the population’s preferences.

## Supporting information

S1 Table PRISMA 2009 checklist. (DOC)

S2 Table Description of attributes and levels by device or HTA. (DOCX)
