# Supplementary material for: What attributes should be included in a discrete choice experiment related to health technologies? A systematic literature review
Source: PLoS One. 2019 Jul 18;14(7):e0219905. doi: 10.1371/journal.pone.0219905 (PMC6639002; doi:10.1371/journal.pone.0219905)
Supplement: S2 Table — (DOCX) [file pone.0219905.s002.docx]

**S2 1. Table. Description of attributes and levels by device or HTA (n=72)**

| **Author(s)** | **Publication year** | **Device/HTA** | **Attributes** | **Levels** |
| --- | --- | --- | --- | --- |
| **Ahmed, S. F., W. A. Smith, et al.** | **2008** | Injection device for growth hormone threapy | 1.Operated by | (2): auto-injector; plunger |
|  |  |  | 2.Weight | (4): like a pencil (10g); like half an apple (50g); like a standard apple (100g); like a large apple (150g); |
|  |  |  | 3.Sound made | (4): none; soft (heard by user only); distinct (heard within 2m); loud (easily heard by all those in same room) |
|  |  |  | 4.Preparation | (2): no mixing required; mixing required weekly-to-monthly |
|  |  |  | 5.Needle | (3): needle-free; 0.5cm needle; 1cm needle |
|  |  |  | 6.Size | (4): 9cmx3cm; 9cmx6cm; 15cmx6cm; 15cmx9cm |
| **Augustovski, F., A. Beratarrechea, et al.** | **2013** | Biological drugs for rheumatoid arthritis | 1. Patient Global Assessment of disease activity (PGA) | (3): 40 mm; 30 mm; 20 mm |
|  |  |  | 2. Mode ofadministration | (3): Oral; Subcutaneous; Intravenous |
|  |  |  | 3. Frequencyofadministration | (4): Every10mo; Everymonth; Everyweek; Everyday |
|  |  |  | 4. Local adverseevents | (3): No risk; 15 patients out of 100; 40 patients out of 100 |
|  |  |  | 5. Generalized adverseevents | (3): No risk; 10 patients out of 100; 30 patients out of 100 |
|  |  |  | 6. Serious infections | (2): 1 patients out of 100; 5 patients out of 100 |
|  |  |  | 7. Costs | (3): No out-of-pocket cost; $500 (Argentinepesos) per month; $1500 (Argentinepesos) per month |
| **Bøgelund, M., T. Vilsbøll, et al.** | **2011** | Diabetes treatment | Convenience attributes |  |
|  |  |  | 1. Mode of administration | (4): injection twice a day in relation to meal; injection once a day in relation to meal; injection once a day irrespective of meals; oral anti-diabetes drugs up to three a day with meals |
|  |  |  | 2. Blood glucose monitoring | (4): three times a day; once a day; once per week; no need for testing |
|  |  |  | 3. Payment per month | (4): DKK 1000 per month; DKK 500 per month; DKK 200 per month; DKK 0 per month |
|  |  |  | Clinical attributes |  |
|  |  |  | 4. Number of hypoglycaemic events | (4): approximately once a week; approximately once a month; approximately once a year; none |
|  |  |  | 5. HbA | (3): approximately 9%; approximately 7.5%; approximately 6.5% |
|  |  |  | 6. Weight | (4): gain 3kg; remain the same weight; lose 3kg; lose 6kg |
|  |  |  | 7. Transient nausea | (2): mild nausea for up to 3 months; none |
|  |  |  | 8. Payment | (4): DKK 2000 per month; DKK 500 per month; DKK 200 per month; DKK 0 per month |
|  |  |  | Other aspects (concomitant diseases) |  |
|  |  |  | 9. Anti-hypertension treatment | (1): one less anti-hypertension medication |
|  |  |  | 10. Blood pressure | (2): blood pressure drop of 5mmHg: blood pressure drop of 10mmHg |
|  |  |  | 11. Heart function | (1): improvement allowing walking twice the distance of current limit |
|  |  |  | 12. Driver's licence | (1): can have a driver's licence |
| **Bonnichsen, O.** | **2011** | Ostomy pouches | 1. Flexibility of the system as a whole | (3): Same as current; Small improvement; Large improvement |
|  |  |  | 2. Number of small starting leakagesunder the base plate per month | (3): 3; 1; none |
|  |  |  | 3. Filter lifetime (hours) | (3): 7; 12; 24 |
|  |  |  | 4. Additional expense per month (SEK) | (7): (0); 125; 200; 375; 500; 750; 1000 |
| **Bridges, J. F. P., E. T. Kinter, et al.** | **2011** | Treatment for schizophrenia | 1. Improved social activity | (2): is better able to interact with other people; has experienced no change in social activity |
|  |  |  | 2. No weight gain | (2): has not gained any weight; has experienced weight gain |
|  |  |  | 3. Improvements in stressful situations | (2): improved ability to deal with a stressful situation; no improvement in their ability to deal with a stressful situation |
|  |  |  | 4. Availability of group therapy | (2): has group therapy available and is easily accessible; group therapy was not available |
|  |  |  | 5. Not feeling slowed | (2): has no change in amount of energy; feels slowed down, like a 'zombie' |
|  |  |  | 6. A supportive physician | (2): was able to discuss treatment objectives and side effects with treating physician; treating physician was not accessible or willing to discuss the new treatment |
| **Bridges, J. F. P., A. T. Lataille, et al.** | **2012** | Hearing aid (how well the hearing aid assisted patients in various listening environments) | 1. Quiet setting | (2): more effective in quiet settings; somewhat effective in quiet settings |
|  |  |  | 2. Comfort | (2): rarely causes physical discomfort; occasional physical discomfort |
|  |  |  | 3. Feedback | (2): occurs 4 times a month; occurs 12 times a month |
|  |  |  | 4. Battery life | (2): replace batteries 2 times a month; replace batteries 4 times a month |
|  |  |  | 5. Purchase cost | (2): costs US$3000; costs US$5000 |
|  |  |  | 6. Water/sweat | (2): somewhat water/sweat resistance; not so water/sweat resistance |
|  |  |  | 7. Noisy settings | (2): more effective in noisy settings; somewhat effective in noisy settings |
| **Bridges, J. F. P., A. F. Mohamed, et al.** | **2012** | Treatment for advanced non-small cell lung cancer | 1. Progression-free survival | (3): 4 months; 5 months; 7 months |
|  |  |  | 2. Symptom severity | (3): mild; moderate; severe |
|  |  |  | 3. Rash | (3): none; mild; moderate |
|  |  |  | 4. Diarrhoea | (3): none; mild; moderate |
|  |  |  | 5. Fatigue | (3): none; mild; moderate |
|  |  |  | 6. Nausea and vomiting | (3): none; mild; moderate |
|  |  |  | 7. Fever and infection | (3): none; fever; infection with fever |
|  |  |  | 8. Mode of treatment administration | (3): oral; infusion; oral and infusion |
| **Bridges, J. F. P., S. C. Searle, et al.** | **2012** | Male circumcision | 1. Follow-up visit after circumcision | (2): yes; no |
|  |  |  | 2. Infection rate following circumcision | (2): 1 in 100; 3 in 100 |
|  |  |  | 3. Number of days of pain following circumcision | (2): 1 day; 4 days |
|  |  |  | 4. Type of waiting room for the circumcision service | (2): wait outside; wait in a private waiting room |
|  |  |  | 5. Counseling on the risks and benefits of circumcision | (2): yes; no |
|  |  |  | 6. Predominant staff | (2): male; female |
|  |  |  | 7. Having links to traditional circumcision schools | (2): yes; no |
|  |  |  | 8. Is a 3-day advance booking required | (2): yes; no |
|  |  |  | 9. How the boy comes to the clinic | (2): individually; in groups |
|  |  |  | 10. HIV test is required | (2): yes; no |
|  |  |  | 11. Predominant clinic staff members | (2): formal; informal |
| **Bunge, E. M., E. W. de Bekker-Grob, et al.** | **2010** | Brace treatment for scoliosis | 1. Reduction in risk of surgical intervention (%) | (4): 12,5; 25; 50; 75 |
|  |  |  | 2. Brace is visible (visible) | (2): yes; no |
|  |  |  | 3. Total treatment duration (yr) (time) | (4): 2; 4; 6; 8 |
|  |  |  | 4. Brace is uncomfortable (discomfort) | (2): yes; no |
| **Burnett, H. F., D. A. Regier, et al.** | **2012** | Drug treatment in juvenile idiopathic arthritis | 1. Child reported pain from arthritis | (4): none; mild; moderate; severe |
|  |  |  | 2. Participation in daily activities | (4): without any difficulty; with some difficulty; with much difficulty; unable to participate |
|  |  |  | 3. Side effects | (4): none; headache 4 days per month; stomach pain 4 days per month; nausea 4 days per month |
|  |  |  | 4. Drug treatment | (3): self- or parent managed pill 4 days per month; self- or parent managed injection 4 days per month; dcotor- or nurse managed IV 1 day per month |
|  |  |  | 5. Days missed from school | (4): half-day per month; one day per month; two days per month; four days per month |
|  |  |  | 6. Cost to you | (4): $50 per month; $700 per month; $1500 per month; $2100 per month |
| **Chancellor, J., M. Martin, et al.** | **2012** | Treatment of chronic pain with classic opioids | 1. Effect of medication on your pain | (3): pain not preventing any usual activities; pain preventing some usual activities; pain preventing basic activities: cannot go out |
|  |  |  | 2. Constipation and bowel problems | (3): none or very slight; feeling bloated, sluggish, uncomfortable; no evacuation for several days, abdominal pain |
|  |  |  | 3. Nausea and vomiting | (3): none or very mild and occasional; nauseous: cannot do usual activities; persistent vomiting and retching |
|  |  |  | 4. Alertness | (3): can concentrate well; forgetful, hard to concentrate; feeling out of control, difficulty talking |
|  |  |  | 5. Energy | (3): not sleepy; feeling sleepy, lazy or apathetic; cannot stay awake, just want to sleep |
| **Damen, T. H. C., E. W. de Bekker-Grob, et al.** | **2011** | Breast reconstruction | 1. Material used fore reconstruction | (3): implant; autologous tissue from abdomen; autologous tissue from back with implant |
|  |  |  | 2. Number and duration of operations | (3): 1x 3h; 2x1h; 1x7h |
|  |  |  | 3. Short-term complication rate | (3): 1%; 5%; 10% |
|  |  |  | 4. Long-term complication rate | (3): 0%; 10%; 30% |
|  |  |  | 5. Aesthetic result | (3): moderate; good; excellent |
|  |  |  | 6. Waiting time | (3): 0 months; 6 months; 12 months |
| **Darbà, J., G. Restovic, et al.** | **2011** | Severe osteoporosis treatment | 1. Type of administration | (3): oral; subcutaneous; intravenous |
|  |  |  | 2. Place of administration | (3): self-administration; medical support; hospitalization |
|  |  |  | 3. Cost | (3): 10€; 50€; 100€ |
| **de Bekker-Grob, E. W., M. L. Essink-Bot, et al.** | **2008** | Preventive drugs for osteoporosis | 1. Route of drug administration | (4): tablet once per month; tablet once a week; injection by GP every four months; injection by GP every month |
|  |  |  | 2. 10-year risk reduction of a hip fracture | (4): 5; 10; 25; 50 |
|  |  |  | 3. Nausea (during two hours after use) | (2): no; yes |
|  |  |  | 4. Total treatment duration | (4): 1; 2; 5;10 |
|  |  |  | 5. Total cost to you (thus per month) | (4): 0; 120; 240; 720 |
| **de Bekker-Grob, E. W., M.-L. Essink-Bot, et al.** | **2009** | Osteoporosis drug treatment | 1. Route drug administration | (4): tablet once a month; tablet once aweek; injection by GP every 4 months; injection by GP every month |
|  |  |  | 2. Effectiveness | (4): 5; 10; 25; 50 |
|  |  |  | 3. Adverse effect | (2): no nausea; nausea |
|  |  |  | 4. Total treatment duration | (4): 1; 2; 5; 10 |
|  |  |  | 5. Total cost to patient | (4): 0; 120; 240; 720 |
| **de Bekker-Grob, E. W., R. Hofman, et al.** | **2010** | HPV Vaccination | 1. Protection against cervical cancer | (3): 50%; 70%; 90% |
|  |  |  | 2. Protection duration | (3): 6years; 25 years; lifetime |
|  |  |  | 3. Serious side-effects | (3):1/750000; 1/150000; 1/30000 |
|  |  |  | 4. Mild side-effects | (3): 1/50; 1/30; 1/10 |
|  |  |  | 5. Age at vaccination | (3): at age 9 years; at age 12 years; at age 14 years |
| **de Bekker-Grob, E. W., L. Hol, et al.** | **2010** | Fecal occult blood test, sigmoidoscopy and colonoscopy for colorectal cancer | 1. Reduction in mortality | (4): from 3% to 0,3%; from 3% to 1,2%; from 3% to 1,8%; from 3% to 2,7% |
|  |  |  | 2. Frequency of screening per 10 years | (5): 0;1;2;5;10 |
|  |  |  | 3. Complication risk | (2): none; small |
|  |  |  | 4. Location of screening | (3): home; hospital; none |
|  |  |  | 5. Screening duration | (5): 0min; 10min; 30min; 60min; 90min |
|  |  |  | 6. Preparation for patient | (5): none; enema; no fasting; drinking 0,75l+fasting; drinking 4l+fasting |
|  |  |  | 7. Side effects of screening | (2): none; mild pain |
| **Deal, K., D. Marshall, et al.** | **2013** | Gastroesophageal reflux disease treatments | 1. Medication cost per month | (3): $3 per month; $30 per month; $50 per month; $70 per month |
|  |  |  | 2. When you take your GERD medication? | (4): twice daily; once daily; 1 pill when you think acid reflux might occur; 1 pill after acid reflux begins |
|  |  |  | 3. Diet changes | (4): must radically change your diet; must eliminate eating some foods and beverages you like; must reduce eating some foods and beverages you like; you are able to eat what you are before GERD; |
|  |  |  | 4. Daytime discomfort due to acid reflux and heartburn | (5): 4 or more episodes reflux during the day; 1 to 3 episodes of acid reflux durign the day; 1 to 3 episodes of acid reflux durign a week; 1 to 3 episodes of acid reflux durign a month; no episodes of acid reflux during the daytime |
|  |  |  | 5. Sleeping discomfort due to acid reflux and heartbun | (5): 4 or more episodes reflux during the night's sleep; 1 to 3 episodes of acid reflux durign the night's sleep; 1 to 3 episodes of acid reflux durign your sleep over a week; 1 to 3 episodes of acid reflux durign your sleep over a month; no episodes of acid reflux during your sleep |
|  |  |  | 6. Side effects | (4): no side effects; mild side effects; moderate side effects; severe side effects |
| **Essers, B. A. B., C. D. Dirksen, et al.** | **2010** | Surgical treatment of primary basal cell carcinoma | 1. Probability of tumour recurrence | (4): 1%; 2%; 3%; 5% |
|  |  |  | 2. Time between start and end of surgery | (4): 44min; 52min; 145min; 165min |
|  |  |  | 3. Time it takes to travel from home to hospital | (3): 15min; 30min; 60min |
|  |  |  | 4. Lenght of time to wait before surgery results available | (3): 0weeks; 1 week; 2 weeks |
|  |  |  | 5. Possibility of having a second excision | (2): no one; 1 in 5 patients |
|  |  |  | 6. Price one has to pay for surgery | (5): 0€;200€;500€;1000€;1500€ |
| **Essers, B. A. B., D. van Helvoort-Postulart, et al.** | **2010** | Surgical treatment of primary basal cell carcinoma | 1. Probability of BCC recurrence (%) | (4): 1; 2; 3; 5 |
|  |  |  | 2. Duration of surgery (minutes) | (4): 44; 52; 145; 165 |
|  |  |  | 3. Probability of having a re-excision (%) | (2): 0; 20 |
|  |  |  | 4. Waiting time for surgery results (weeks) | (3): 0; 1; 2 |
|  |  |  | 5. Travel time to hospital (minutes) | (3): 15; 30; 60 |
|  |  |  | 6. Costs of surgery (h) | (5): 0; 200; 500; 1000; 1500 |
| **Fegert, J. M., L. Slawik, et al.** | **2011** | Tdrug treatment for attention-deficit/hyperactivity disorder of school age children | 1. Duration of action | (2): long (whole day); short (half day) |
|  |  |  | 2. Side effects | (2): none; potential weight loss |
|  |  |  | 3. Dosage/product | (2): variable, compatible (combinations are possible); specified |
|  |  |  | 4. Discretion | (2): Discrete use of medication; Medication cannot be used discretely |
|  |  |  | 5. Emotional situation | (2): No mood swings; Mood swings may occur |
|  |  |  | 6. Social situation | (2): Opportunities for friendships and hobbies; Impairment with regard to friendships and hobbies |
|  |  |  |  |  |
| **Golan, O., Hansen, P., et al** | **2010** | Health technologies | 1. Lives saved, including ‘statistical’ lives | (5): none; few; some; many; very many |
|  |  |  | 1. Life prolongation benefits – in terms of increase in life expectancy and its quality-of-life, and number of patients affected | (4): none/very small; small benefits; medium benefits; large benefits |
|  |  |  | 1. Quality-of-life gains | 4): none/very small; small QoL gains; medium QoL gains; large QoL gains |
|  |  |  | 1. If this technology were not to be funded… | (3): Many/most patients will be able to pay for it themselves; many most patients will get an alternative treatment (less effective) already funded by the government; many/most patients will not receive any treatment for condition |
|  |  |  | 1. Other important social ethical benefits | (2): none/very small; yes |
| **Green, C. and K. Gerard** | **2009** | Health technologies | 1. Severity | (2): yes; no |
|  |  |  | 2. Health improvement | (4): large; moderate; small; very small |
|  |  |  | 3. Value for money | (4): very good; fairly good; fairly poor; very poor |
|  |  |  | 4. Other treatment | (2): yes; no |
| **Grutters, J. P. C., A. G. H. Kessels, et al.** | **2008** | Hearing aid | 1. Performer and accuracy of initial assessment | (4): ENT specialist at hospital; dispenser at practice equally accurate; dispenser at practice 10% less accurate; dispenser at practice 20% less accurate |
|  |  |  | 2. Duration of hearing aid provision | (4): 2 months; 4 months; 6 months; 8 months |
|  |  |  | 3. Follow-up at ENT specialist | (2): yes; no |
|  |  |  | 4. Discount for WTA/extra payment for WTP | (4):no; 20€; 50€; 100€ |
| **Guo, N., C. A. Marra, et al.** | **2011** | Latent tuberculosis infection preventive treatment | 1. Lenght of treatment | (4): 4 months; 6 months; 9 months; 12 months |
|  |  |  | 2. Frequency of clinic visit | (3): every 2 months; every 1 month; every 2 weeks |
|  |  |  | 3. Risk of active TB developing after treatment | (4): 0%; 1%; 2%; 4% |
|  |  |  | 4. Chance of liver damage developing | (5):0%; 1%; 3%; 5%; 10% |
|  |  |  | 5. Chance of skin rash developing | (3): 0%; 5%; 10% |
|  |  |  | 6. Chance of fatigue developing | (3): 0%; 5%; 10% |
| **Hauber, A. B., N. K. Arden, et al.** | **2013** | Non-selective nonsteroidal anti-inflammatory drugs (NSAIDs) and COX-2 inhibitors | 1. Pain while moving around | (4): none; mild; moderate; severe |
|  |  |  | 2. Pain while sitting, lying down, or sleeping 1 h after taking the medicine | (4): none; mild; moderate; severe |
|  |  |  | 3. Stiffness 1h after taking the medicine | (4): none; mild; moderate; severe |
|  |  |  | 4. Difficulty doing your daily activities 1h after taking the medicine | (4): none; mild; moderate; severe |
|  |  |  | 5. Chance of a bleeding ulcer | (4): none; 10 out of 1000; 50 out of 1000; 100 out of 1000 |
|  |  |  | 6. Additional chance of a heart attack or stroke | (4): no chance; 5 out of 1000; 15 out of 1000; 30 out of 1000 |
| **Herbild, L., M. Bech, et al.** | **2009** | Pharmacogenetic testing in the treatment for depression | 1. Number of changes in antidepressants before symptom relief | (2): 2 changes; 3 changes |
|  |  |  | 2. Time with dosage adjustments due to adverse side effects and/or lack of effects | (2): 1 month; 3 months |
|  |  |  | 3. Cost of pharmacogeneti testing | (8): DKK200; DKK600; DKK1000; DKK1500; DKK3000; DKK6000; DKK9000; DKK18000 |
|  |  |  | 4. Probability of benefits from pharmacogenetic testing | (2): 10%; 50% |
| **Hill, M., J. Fisher, et al.** | **2012** | Prenatal tests for Down syndrome | 1. Accuracy | (3): 90%; 95%; 100% |
|  |  |  | 2. Time of results (gestation in weeks) | (3):9;11;13 |
|  |  |  | 3. Risk of miscarriage | (2): small risk; no risk |
|  |  |  | 4. Information gained from the test | (2): down syndrome; down syndrome plus additional information |
| **Hinoul, P., Goossens, A., et al.** | **2010** | Mesh technique to treat urogenital prolapse | 1. Current level of scientific evidence for the novel procedure | (2): high level; low level |
|  |  |  | 2. Post-operative pain | (2): little post-operative pain; severe post-operative pain |
|  |  |  | 3. Exposure risk of the mesh | (2): low risk of exposure (≤5); high risk of exposure (>5%) |
|  |  |  | 4. Required surgical skill | (2): easy procedure; difficult procedure |
|  |  |  | 5. Duration of hospital admission | (2): short (defined as ≤3 days); long (defined as >3 days) |
|  |  |  | 6. Cost price | (2) inexpensive; expensive |
| **Hodgkins, P., P. Swinburn, et al.** | **2012** | Oral Treatment for Mild-to-Moderate Ulcerative Colitis | 1. Ease of swallowing | (3): No problems swallowing; Slight problems swallowing; Some problems swallowing |
|  |  |  | 2. Number of doses/Time of day | (3): Only in the morning or only in the evening; In the morning and in the evening; In the morning, afternoon and evening |
|  |  |  | 3. Quantity of medication needed | (3): one; two; three |
|  |  |  | 4. Treating your symptom flare | (3): Improvement in symptoms (Fewer stools and/or less bleeding); Return to normal stool frequency and no bleeding; Return to normal stool frequency, no bleeding and some healing of the inner lining of your bowel |
|  |  |  | 5. Risk of symptom flares | (3): 10% (1 in 10 patients will experience a flare within the next 12 months); 40% (4 in 10 patients will experience a flare within the next 12 months); 70% (7 in 10 patients will experience a flare within the next 12 months) |
|  |  |  | 6. Cost | (3): You pay £20 per month; You pay £40 per month; You pay £60 per month |
| **Hol, L., E. W. De Bekker-grob, et al.** | **2010** | Colorectal cancer screening test | 1. Screening interval FOBT | (3):1/3;1;3 |
|  |  |  | 2. Screening interval Sigmoidoscopy | (3):1;5;10 |
|  |  |  | 3. Screening interval Colonoscopy | (3):2;5;10 |
|  |  |  | 4. Risk reduction FOBT | (3):10;25;40 |
|  |  |  | 5. Risk reduction Sigmoidoscopy | (3):40;50;70 |
|  |  |  | 6. Risk reduction Colonoscopy | (3):75;85;95 |
| **Howard, K. and G. Salkeld** | **2009** | Colorectal cancer screening test | 1. Accuracy test: how many cancers the test will find | (4): 55/100 cancers; 65/100 cancers; 75/100 cancers; 85/100 cancers |
|  |  |  | 2. Accuracy test: how many cancers the test will miss? | (4):45/100 cancers; 35/100 cancers; 25/100 cancers; 15/100 cancers |
|  |  |  | 3. Accuracy test: how many large polyps the test will find | (4): 35/100 polyps; 45/100 polyps; 55/100 polyps; 65/100 polyps |
|  |  |  | 4. Accuracy test: how many large polyps the test will miss? | (4): 65/100 polyps; 55/100 polyps; 45/100 polyps; 35/100 polyps |
|  |  |  | 5. Accuracy test: number of people who are correctly reassured by the test that they do not have cancer | (4): 800/1000 people; 850/1000 people; 900/1000 people; 950/1000 people |
|  |  |  | 6. Accuracy test: numer of people who have unnecessary colonoscopies | (4): 60/1000 people; 80/1000 people; 100/1000 people; 120/1000 people |
|  |  |  | 7. Cost | (4):$20;$30;$40;$50 |
|  |  |  | 8. Dietary and medication restrictions | (1):no |
|  |  |  | 9. How the sample is collected | (1): brush stool surface gently then dab on test kit |
| **Howard, K., G. Salkeld, et al.** | **2011** | Colonography and colonoscopy as diagnostic tests | 1. How likely it is that you will need a second procedure after CTC to treat polyps or cancer? | (2): 200/1000 people; 400/1000 people |
|  |  |  | 2. Bowel preparation | (2): intensive; minimal |
|  |  |  | 3. Test accuracy (likely of missing small cancers or polyps) | (2): 20/1000 people tested; 40/1000 people tested |
|  |  |  | 4. The one-off cost to you personally | (4): AUS$0 (no cost);AUS$100; AUS$200; AUS$300 |
| **Ijzerman, M. J., J. A. van Til, et al.** | **2012** | Treatment Alternatives for Stroke Rehabilitation | 1. Clinical outcome | (9): 1 (the criteria are equally important) to 9 (‘‘extremely more important’’ ) |
|  |  |  | 2. Probability of outcome | (9): 1 (the criteria are equally important) to 9 (‘‘extremely more important’’ ) |
|  |  |  | 3. Easy of use | (9): 1 (the criteria are equally important) to 9 (‘‘extremely more important’’ ) |
|  |  |  | 4. Cosmetics and confort | (9): 1 (the criteria are equally important) to 9 (‘‘extremely more important’’ ) |
|  |  |  | 5.Complication type | (9): 1 (the criteria are equally important) to 9 (‘‘extremely more important’’ ) |
|  |  |  | 5. Probability of complication | (9): 1 (the criteria are equally important) to 9 (‘‘extremely more important’’ ) |
|  |  |  | 6. Treatment duration | (9): 1 (the criteria are equally important) to 9 (‘‘extremely more important’’ ) |
|  |  |  | 8. Invasiveness of treatment | (9): 1 (the criteria are equally important) to 9 (‘‘extremely more important’’ ) |
| **Johnson, F. R., A. B. Hauber, et al.** | **2010** | Irritable bowel syndrome | 1. Frequency of abdominal pain and discomfort | (5): no IBS pain and discomfort; IBS pain and discomfort 1 week/month; IBS pain and discomfort 2 weeks/month; IBS pain and discomfort 3 weeks/month; IBS pain and discomfort 4 weeks/month; |
|  |  |  | 2. Diarrhea frequency | (4): no diarrhea; diarrhea 2 times/day; diarrhea 4 times/day; diarrhea more than 4 times/day |
|  |  |  | 3. Urgency frequency | (4): no urgency; urgency 2 days/week; urgency 5 days/week; urgency 7 days/week |
|  |  |  | 4. Frequency of mild-to-moderate constipation | (5): no constipation; constipation 1 week/month; constipation 2 weeks/month; constipation 3 weeks/month; constipation 4 weeks/month; |
|  |  |  | 5. Chance of serious adverse event | (13): no chance of severe adverse events; 1/1000 person will have moderate colitis requiring doctor's care; 5/1000 people will have moderate colitis requiring doctor's care; 10/1000 will have moderate colitis requiring doctor's care; 1/1000 will have an impacted bowel requiring doctor's care; 5/1000 people will have an impacted bowel requiring doctor's care; 10/1000 people will have an impacted bowel requiring doctor's care; 1/1000 will have severe colitis requiring hospitalization; 5/1000 will have severe colitis requiring hospitalization; 10/1000 will have severe colitis requiring hospitalization; 1/1000 will have a perforated bowel requiring surgery; 5/1000 will have a perforated bowel requiring surgery; 10/1000 will have a perforated bowel requiring surgery |
| **Kolasa, K., Zwolinski K.M., et al** | **2016** | Orphan drugs | 1. Indication uniqueness 2. Disease rarity 3. Disease severity 4. Advancement of technology 5. Manufacturing technology complexity 6. Therapeutic alternative (unmet medical need) 7. Scientific evidence for clinical efficiency (level of uncertainty) 8. Benefits from use of medicine (safety and   adverse effects)   1. Cost effectiveness 2. Budget impact (in €) | (3): one unique indication; more tan one orphan indications; and, one or more indications for common disease  (3): prevalence <0.5 per 10,000 UE citizens; prevalence in the range of 0.5 and 1 per 10,000 UE citizens; and, prevalence >1 per 10,000 UE citizens  (3): high mortality often with poor prognosis; chronic without high mortality and morbidity; and, severe invalidity, severely harm of capacities central to individuals’ functioning in society  (3): Advanced therapy medicinal product (ATMP) including biopharmaceutical, innovative  synthetic entities and delivery systems as well non-biological complex drug; conventional small molecule (molecular weight, MW< 500 Da) with at least one stereogenic (chiral) center in its structure; and, widely available simple chemical entities (e.g. zinc acetate) including molecules easily synthesized from commercially available precursors - fine chemicals  (3): expensive biotechnological processes; complex synthetic path consisting of at least three independent chemical  Transformations; and, manufacturing require the use of separation techniques for most intermediates  (3): no comparable alternative available; second line treatment available; and, at least one comparable alternative available  (4): randomised placebo(or active)-controlled clinical trial(s) (RCT) with hard endpoints  such as overall survival or time to progression (TTP); randomised placebo(or active)-controlled clinical trial(s) (RCT) with surrogate endpoints; uncontrolled non-randomised clinical trial(s), observation studies or cohort studies with; and, very limited data with high level of uncertainty – e.g. case reports  relevant but only limited level of uncertainty  (3): Only minor and reversible adverse events; only minor and reversible adverse events; and, high incidence of severe of adverse events  (3): ICER below 24 k€; ICER in the range between 24 k€ and 48 k€; and, ICER above 48 k€  (3): budget savings or positive budget impact below 1,2 M€; in the range between 1,2 and 2,4 M€; and, above 2,4 M€ |
| **Kinter, E. T., T. J. Prior, et al.** | **2012** | Treatment for schizophrenia patients | 1. Disease symptoms | (2): patient was able to manage while experiencing disease symptoms (e.g., delusions, hostility, depression); patient was not patient able to manage while experiencing disease symptoms (e.g., delusions, hostility, depression). |
|  |  |  | 2.Relapse | (2): there was a clear worsening of disease symptoms that caused the patient to be treated, and stabilized, in an outpatient setting; there was not a clear worsening of disease symptoms that caused the patient to be treated, and stabilized, in an outpatient setting |
|  |  |  | 3.Clear thinking | (2): the patient was able to think clearly and have control over their thoughts; the patient was not able to think clearly and have control over their thoughts |
|  |  |  | 4. Social activities | (2): the patient found it difficult to participate in social activities (e.g., time for family, friends or going to the movies); the patient did not find it difficult to participate in social activities (e.g., time for family, friends or going to the movies) |
|  |  |  | 5. Extrapyramidal symptoms | (2): the patient had EPS (e.g., inertness, restlessness in the legs, salivation) that limited their body movements; the patient had not EPS (e.g., inertness, restlessness in the legs, salivation) that limited their body movements |
|  |  |  | 6. Daily activities | (2): the patient could participate in daily activities (e.g., completing household activities and capable of full-/part-time work); the patient could not participate in daily activities (e.g., completing household activities and capable of full-/part-time work) |
|  |  |  | 7. Support | (2): the patient’s physician was also looking after the patient’s personal needs and well-being, in addition to looking after the patient’s medical needs; the patient’s physician was not looking after the patient’s personal needs and well-being, in addition to looking after the patient’s medical needs. |
| **Koopmanschap, M. A., Stolk, E.A. and Koolman,X.** | **2010** | Unlabeled curative treatments | 1. National additional medical costs per year (budget impact) | (3): 10 million €; 20 million €; 50 million €; |
|  |  |  | 2. National saving in costs of absence from work per year | (3): 0 million €; 2 million €; 4 million €; |
|  |  |  | 3. Disease severity (before treatment) | (3): low; moderate; high |
|  |  |  | 4. Incremental cost-effectiveness ratio of the intervention | (3): 15000 € per QALY; 45000 € per QALY; 90000 € per QALY; |
|  |  |  | 5. The number of QALYs gained per patient | (3): 0.5 QALYs; 2 QALYs; 4 QALYs |
|  |  |  | 6. The composition of the health gain | (3): 100% longer life; 100% improved QoL; 50% of each |
|  |  |  | 7. The probability that costs per QALY will be at least doubled as compared to the average cost-effectiveness ratio as mentioned above | (3): 10%; 20%; 30% |
| **Kruijshaar, M. E., M.-L. Essink-Bot, et al.** | **2009** | Tests for Barrett esophagus | 1. Type of test | (2): endoscopy; two less burdensome fictitious tests |
|  |  |  | 2. Frequency of surveillance | (5): 2 times; 3 times; 5 times; 10 times; 40 times) |
| **Lathia, N., P. K. Isogai, et al.** | **2013** | Febrile neutropenia | 1. Out-of-pocket costs | (4): $10; $25; $50; $100 |
|  |  |  | 2. Unpaid caregiver time required daily | (4): 1h; 2h; 4h; 8h |
|  |  |  | 3. Probability of return to the hospital | (4): 5%; 10%; 20%; 50% |
| **Laver, K., J. Ratcliffe, et al.** | **2011** | Therapy tool for hospitalised older people | 1. Mode of therapy | (2): conventional therapy; therapy using the Nintendo Wii; |
|  |  |  | 2. Difficulty of therapy | (2): easy (30 min of light activity); challenging (1h moderate activity) |
|  |  |  | 3. Cost | (3): no cost; $25/week; $50/week |
|  |  |  | 4. Amount of recovery made | (3): 70%; 80%; 90% |
| **Lee,W.C., Joshi, A.V., et al.** | **2008** | Haemophilia treatments | 1. Risk of human viral infections | (2): recombinant concentrate; very pure concentrate extracted from human plasma |
|  |  |  | 2. Possibility that the titre of inhibitor may rise | (2): yes; no |
|  |  |  | 3. Reduces the likelihood of dose-related | (2): yes; no |
|  |  |  | 4. Number of infusions required to stop a haemorrhage | (3): 1; 2; 3 |
|  |  |  | 5. Time required to prepare the infusion | (4): ≤5 min; 6-10 min; 11-30 min; >30 min |
|  |  |  | 6. Infusion time | (4): ≤5 min; 6-10 min; 11-30 min; >30 min |
|  |  |  | 7. Infusion volume | (4): ≤15 mL; 16-40 mL; 41-80 mL; 81-120 mL |
|  |  |  | 8. Time required to stop bleeding | (4): ≤6h; 7-12h; 13-24h; >24h |
|  |  |  | 9. Time required to alleviate pain | (4): ≤2h; 3-6h; 7-9h; 10-12h |
|  |  |  | 10. Use in prophylaxis | (2): one infusion every 2 days; one infusion every day |
|  |  |  | 11. Ability to undergo major surgery | (2): yes; no |
|  |  |  | 12. Cost of medications | (3): cost is not relly a consideration; cost is somewhat of a consideration; cost is very much a consideration |
| **Manjunath, R., J.-C. Yang, et al.** | **2012** | Antiepileptic drugs | 1. Number of seizures | (4): 0%; 75%; 50%;25% fewer seizures |
|  |  |  | 2. Duration of short-term side effects | (3): none; las for 1 week; last for 4 weeks |
|  |  |  | 3. Long-term fatigue or modiness | (2): none; cause some difficulty doing daily activities |
|  |  |  | 4. Long-term confusion or memory problems | (2): none; cause some difficulty doing daily activities |
|  |  |  | 5. Difficulty urinating | (3): none; half the time; almost always |
|  |  |  | 6. Weight change in 6 months | (3): 15-pound weight loss; no weight loss or gain; 15-pound weight gain |
|  |  |  | 7. How often you take the medicine? | (3): once a day; twice a day; 3 times a day |
|  |  |  | 8. Personal medicine cost | (5): $0;$25;$75;$150;$300 |
| **Marshall, D. A., F. R. Johnson, et al.** | **2009** | Colorectal cancer screening test | 1. How is it done? | (4): stool; scope; barium enema; CT |
|  |  |  | 2. Is there pain or discomfort? | (3); none; mild;sedative |
|  |  |  | 3. How often will the screening test be done? | (4): once; every 10 years; every 5 years; annual |
|  |  |  | 4. What do I do to prepare? | (4): diet; laxative;enema; none |
|  |  |  | 5. If this screening test result is abnormal will an additional test be needed to confirm whether you have cancer? (follow-up) | (2): none; yes |
|  |  |  | 6. If 10 people without cancer get this screening test, how many of them will the test say do have cancer? (specificity) | (3): 50%; 80%; 90% |
|  |  |  | 7. If 10 people with cancer get this screening test, how many of them will the test say do not have cancer? (sensitivity) | (3): 40%; 70%; 90% |
|  |  |  | 8. How many people who get this screening test have a complication (complication risk)? | (4): none; 1/100; 1/1000;1/10000 |
|  |  |  | 9. What is the cost of the screening test to me(cost)? | (4): $25; $100; $500; $1000 |
| **McTaggart-Cowan, H. M., P. Shi, et al.** | **2008** | Asthma-related treatment | 1. Number of symptom-free days per month | (4): 10;15;20;25 or more |
|  |  |  | 2. Daily dosage of medication | (4): as-needed; 1; 2; 3 or more |
|  |  |  | 3. Out-of-pocket cost per month | (4): 20;40;80;160 |
|  |  |  | 4. Number of thrush episodes per year | (4): none; 1;2;3 |
|  |  |  | 5. Number of tremors/palpitation episodes per month | (4): none; 1;2;3 or more |
|  |  |  | 6. Number of inhalers used daily | (3): 1;2;3 |
| **Mentzakis, E., P. Stefanowska, et al.** | **2011** | Drugs to treat orphan diseases | 1. The cost of treating a single patient | (4): $12,000; $15,000; $50,000; $100,000 |
|  |  |  | 2. The total cost of funding the drug program | (4): $5 million total cost; $10 million total cost; $20 million total cost; $50 million total cost |
|  |  |  | 3. The severity of the disease without treatment | (2): Serious impact; moderate impact |
|  |  |  | 4. The impact of drug treatment on a patient’s health/life-years gained (LYG) | (4): 15 years; 10 years; 5 years; 1 year |
|  |  |  | 5. The cost of treating a single patient (CP) | (4): $1000; $5000; $10,000; $12,000 |
|  |  |  | 6. The total cost of funding the drug program | (4): $50 million total cost; $100 million total cost; $150 million total cost; $200 million total cost |
|  |  |  | 7. The severity of the disease without treatment | (2): Serious impact; moderate impact |
|  |  |  | 8. The impact of drug treatment on a patient’s health/life 15 years 15 years gained ( | (4): 15 years; 10 years; 5 years; 1 year |
| **Mohamed, A. F., V. Kilambi, et al.** | **2012** | Immunoglobulin treatments | 1. How you (your child) take(s) each treatment (mode of administration)? | (2): self-administer; appointment with a healthcare professional |
|  |  |  | 2. How often (your child) take(s) each treatment (frequency)? | (3): once a month; twice a month; four times a month |
|  |  |  | 3. Where you (your child) take(s) the treatment (location)? | (2): home; doctor's office, hospital or clinic |
|  |  |  | 4. Number of needle sticks per treatment | (3); 1;2;4 |
|  |  |  | 5. Time to take each treatment (treatment duration) | (3); 2h; 4h;6h |
| **Mohamed, A. F., Johnson, F.R., et al.** | **2012** | Chronic hepatitis B treatments | 1. How long the medication has been studied (weight of evidence) | (3): 6 years; 3 years; 1 year |
|  |  |  | 2. Probability that the patient's viral load remains undetectable for 5 years, with possible histological improvement or reversal of disease progression | (3): 95 out of 100 (95%); 80 out of 100 (80%); 70 out of 100 (70%); |
|  |  |  | 3. 5-year treatment-related risk of a fracture | (4): none; 1 out of 100 (1%); 5 out of 100 (5%); 10 out of 100 (10%); |
|  |  |  | 4. 5-year treatment-related risk of renal insufficiency, where a fracture has not been detected yet | (4): none; 1 out of 100 (1%); 5 out of 100 (5%); 10 out of 100 (10%); |
|  |  |  | 5. Personal cost to the patient each month | (4): €0; €10; €25; €75 or €150 |
| **Morel T., Aymé S., et al.** | **2016** | Medicines in rare diseases | 1. Chance that the medicine will work 2. Expected health improvement 3. Risk of moderate side effects 4. Risk of serious side effects 5. Treatment duration 6. Burden of treatment 7. Ability to conduct usual activities while on treatment | (3): 10%; 45%; and, 80%  (3): not worse; health improvements; and disease-free  (3): 80%; 30%; and, 10%  (3): 30%; 15%; and, 1%  (3): whole life; 12-24 months; and, <6 months  (3): inpatient care, monthly; outpatient care, weekly; and, home, daily  (3): unable; some problem; and, no problem |
| **Mühlbacher, A. C., I. Rudolph, et al.** | **2009** | Attention-Deficit/Hyperactivity Disorder treatment | 1. Duration of effect | (2): long (all day); short (half day) |
|  |  |  | 2. Side effects | (2): loss of weight occurs; none |
|  |  |  | 3. Dosage/dosage form | (2): always the same; variable, combinable |
|  |  |  | 4. Discretion | (2): intake of drug obvious; intake of drug not obvious |
|  |  |  | 5. Emotional state | (2): mood swings may occur; no mood swings |
|  |  |  | 6. Social situation | (2): no problems with friends, hobbies; problems with friends, hobbies |
| **Opuni, M., D. Bishai, et al.** | **2010** | Antiretroviral therapy | 1. Price per month | (5): ZAR50; ZAR400; ZAR600; ZAR800; ZAR1200 |
|  |  |  | 2. Waiting time | (3): 30min; 2h; 5h |
|  |  |  | 3. HIV clinic branding | (3): not branded as HIV clinic in any way; discritely branded as HIV clinic; clearly branded as HIV clinic |
|  |  |  | 4. Staff attitude | (3): kind, respectful sympathetic; indifferent, enither kind nor rude; rude, disrespectful, unsympathetic |
| **Palumbo, A., P. De La Fuente, et al.** | **2011** | Infertility treatment (ovarian stimulating hormones) | 1. Safety | (2): safer; less safe |
|  |  |  | 2. Effectiveness | (2): more effective; less effective |
|  |  |  | 3. Administration of treatment | (2): more friendly administration of treatment; less friendly administration of treatment |
|  |  |  | 4. Price | (4): <€1000; €1000€1500; €1500-€2000; <€2000 |
|  |  |  | 5. Patient-doctor information sharing | (2): your physician informs you about possible alternative treatments; your physician does not inform you about possible alternative treatments |
| **Pereira, C.C.A, Mulligan, M., et al.** | **2011** | Injectable influenza vaccine | 1. Speed | (2): 60 sec; 90 sec |
|  |  |  | 2. Thimerosal | (2): thimerosal; no thimerosal |
|  |  |  | 3. Contamination risk | (2): high; low |
|  |  |  | 4. Storage | (2): large; small |
|  |  |  | 5. Preparation steps | (2): many; few |
|  |  |  | 6. Dosing errors | (2): high probability; low pobability |
|  |  |  | 7. Price | (2): $10.00 per dose; $12.50 per dose |
| **Petrou, S. and E. McIntosh** | **2009** | Surgical and medical management for miscarriage | 1. Time spent at hospital receiving treatment | (3): overnight; half a day; 1 day |
|  |  |  | 2. Level of pain experienced | (3): low; moderate; severe |
|  |  |  | 3. Number of days bleeding after treatment | (3): 3 days; 8 days; 14 days |
|  |  |  | 4. Time taken to return to normal activities after treatment | (4): 1-2 days; 3-4 days; 5-6 days; 7 days or more |
|  |  |  | 5.Cost of treatment to women | (3): £50; £150; £250 |
|  |  |  | 6. Chance of complications requiring more time or readmission to hospital | (3): very unlikely; quite unlikely; unlikely |
| **Pieterse, A. H., A. M. Stiggelbout, et al.** | **2010** | Treatment with radiotherapy | 1. Survival | (2): 40% (prob with surgery); 60% prob with PRT+surgery |
|  |  |  | 2. Local control | (2): 56% (prob with surgery); 68% prob with PRT+surgery |
|  |  |  | 3. Sexual dysfunction | (2): 15% (prob with surgery); 22% prob with PRT+surgery |
|  |  |  | 4. Incontinence | (2): 89% (prob with surgery); 89-100% prob with PRT+surgery |
| **Pignone, M. P., A. T. Brenner, et al.** | **2012** | Colorectal cancer screening test | 1. Ability to reduce colorectal incidence and mortality | (3): 40%; 60%; 80% |
|  |  |  | 2. Discomfort | (2): mild; moderate |
|  |  |  | 3. Nature of test | (3): no prep time, home test, no recovery time; 1/2 day prep time, test in medical facility, 1h recovery; 1 day prep time, test in medical facility; 24h recovery |
|  |  |  | 4. Frequency | (3): every year; every 5 years; every 10 years |
|  |  |  | 5. Risk of major complications over 10 years | (3): 1/10000; 10/10000;50/10000 |
|  |  |  | 6. Out of pocket costs over 10 years | (3): $100; $250; $500 |
| **Pisa, G., S. Freytag, et al.** | **2013** | Treatment for COPD | 1. Dyspnea | (5): Never dyspnea, except on strong exertion; Dyspnea on exertion; Dyspnea at normal walking pace; Dyspnea on slight effort; Dyspnea even at rest |
|  |  |  | 2. Performance capability (bodily resilience) due to COPD | (5): No restriction; Slight restriction; Moderate restriction; Strong restriction; Very strong restriction |
|  |  |  | 3. Sleep quality due to COPD | (4): Sleep through; Awake rarely; Awake often; Awake very often |
|  |  |  | 4. Onset of action of the medication | (4): Within 5 minutes; Within 10 minutes; Within 15 minutes; Within 30 minutes |
|  |  |  | 5. Frequency of administration of the medication | (2): 1 x daily; 2 x daily |
|  |  |  | 6. Health state after awakening (day start) due to COPD | (5): Normal (no impairment); Slight impairment; Moderate impairment; Strong impairment; Very strong impairment |
|  |  |  | 7. Emotional state due to COPD base medication (no emergency spray) | (2): Permanently worried that the efficacy of the medication decreases during the day; Good feeling that the efficacy of the medication remains the same during the day |
| **Regier, D. A., C. Diorio, et al.** | **2012** | Antibiotic prophylaxis in pediatric oncology | 1. Chances of infection | (4): 10;30;50;70 |
|  |  |  | 2. Chances of death | (4): 0.1;1;3;5 |
|  |  |  | 3. Chances of side effects | (4): 5;15;25;50 |
|  |  |  | 4. Route of administration | (3): 1 oral OD; 1 oral BID; 2 oral: 1 TID and 1 BID |
|  |  |  | 5. Cost of pharmacotherapy | (4):$100; $200; $400; $700 |
| **Ryan, M. and V. Watson** | **2009** | Chlamydia screening test | 1.Place of screening | (4): family planning clinic; genito-urinary medicine clinic; your general practitioner; at home |
|  |  |  | 2. Type of screening test | (3): full pelvic examination; perineal swab; urine test |
|  |  |  | 3. Cost to you of screening test | (4): £0; £5;£10; £25 |
|  |  |  | 4. Risk of pelvic inflammatory disease (PID) if you have Chlamydia and are not treated | (4): 0%; 5%, 10%; 25% |
|  |  |  | 5. Support of a trained health-care advisor when you receive you test results | (2): yes; no |
| **Ryan, M., V. Watson, et al.** | **2009** | Bowel cancer screening test | 1. How long people must restrict their diet for before having the screening test | (3):1;2;3 |
|  |  |  | 2. How likely it is that the tests will suggest that someone has cancer when in fact the person does not | (3): 8//1000; 15/1000;20/1000 |
|  |  |  | 3. The cost of the test | (3): £16; £32; £48 |
|  |  |  | 4. Whether people are told when their test results are normal | (3): yes; no |
| **Sadique, M. Z., N. Devlin, et al.** | **2013** | Vaccination against infection diseases | 1. Vaccination | (3): rotavirus (mild infection); invasive pneumococcal disease (severe but rare disease); non-invasive pneumococcal disease (disease with moderate incidence and severity) |
|  |  |  | 2. No vaccination | (3): rotavirus (mild infection); invasive pneumococcal disease (severe but rare disease); non-invasive pneumococcal disease (disease with moderate incidence and severity) |
| **Sassi, F. And McKee, M.** | **2008** | Diagnostic test | 1. Positive predictive value (proportion of those with a positive test who are suitable for surgery) | (3): 90%; 98%; 100% |
|  |  |  | 2. Negative predictive value (proportion with a negative test unsuitable for surgery) | (3): 90%; 98%; 100% |
|  |  |  | 3. Risk of a serious adverse outcome | (2): 0%; 1% |
|  |  |  | 4. Innovative technologies | (2): innovative technology; established technology |
| **Shafey, M., S. M. Lupichuk, et al.** | **2011** | Treatment for relapsed follicular lymphoma (chemotherapy and radioimmunotherapy) | 1. Administration of treatment | (4):outpatient and oral treatment every 28 days x 6 cycles; outpatient day 1 and day 8 only; hospitalization with treatment through central line x 6 days; hospitalization treatment through central line x 6 days immunosuppressive medications |
|  |  |  | 2. Survival free of relapse side effects | (2): 10-15%; 50% |
|  |  |  | 3. Side effects | (4): 1/5 hospitalized; 1/10 hospitalized; 3 weeks in hospital; 4 weeks in hospital |
|  |  |  | 4. Health cost | (4): $5000; $25000; $50000; $150000 |
| **Skjoldborg, U. S., J. Lauridsen, et al.** | **2009** | Treatment for rheumatoid arthritis (TNF-alpha blockers) | 1. Duration of morning stiffness | (6):0;5;30;60;90;120 |
|  |  |  | 2. Pain level | (6):0;2;4;6;8;10 |
|  |  |  | 3. Number of swallen points | (6):0;5;10;15;20;25 |
|  |  |  | 4. Feeling of being tired | (2): reduced (0); unchanged (1) |
|  |  |  | 5. Slightly higher risk of a minor infection | (2): yes; no |
|  |  |  | 6. Out of pocket payment per month in excess of present expenditure for arthritis medication | (18):0;50;100;200;450;575;800;900;1075;1150;1250;1500;2150;2300;2500;3000;4300;5000 |
| **Stockwell, M. S., S. L. Rosenthal, et al.** | **2011** | HPV Vaccines | 1. Mode of transmission of infection | (2): sexually transmitted or not; |
|  |  |  | 2. Vaccines efficacy | (3): 50%; 70%; 90% |
|  |  |  | 3. Severity of the infection | (3): curable; chronic; fatal |
|  |  |  | 4. Behavioural strategy to prevent the infection | (2): hand washing for non-STI and condom use for STI |
| **Sussex J., Rollet, P., et al.** | **2013** | Orphan medicines | 1. Availability of effective existing treatment options 2. Main target patient outcome 3. Therapeutic area 4. Prevalent population range in the European Union 5. Pivotal trial-data package | (2): yes; no  (2): survival; and, progression of disease  (2): immunodeficiency; and, neuromuscular  (2): 250-600 patients; and 3000-8000 patients  (2): open clinical trial (no control group); and, randomized double-blind placebo controlled |
| **Sweeting, K. R., J. A. Whitty, et al.** | **2011** | Treatment of Achilles Tendon Pain | 1. Out-of-pocket cost of treatment ($A) | (3): 1; 240; 480 |
|  |  |  | 2. Treatment type | (3): Exercises; Injections; Exercises and injections |
|  |  |  | 3. Chance of success after 12 weeks (%) | (3): 60; 70; 80 |
|  |  |  | 4. Time before being able to exercise pain-free (wks) | (3): 6; 12; 18 |
|  |  |  | 5. Chance of minor side effects | (3): 1 in 2; 1 in 4; 1 in 6 |
| **Torbica, A. And Fattore, G.** | **2010** | Treatment to reduce cardiovascular risk | 1. Quality of clinical evidence | (3): evidence obtained from three RCTs, all three of which are favorable for the treatment; evidence obtained from one large RCT; evidence obtained from one small RCT |
|  |  |  | 2. Size of health gain | (2): Relative risk reduction of 20%; relative risk reduction of 5% |
|  |  |  | 3. Economic impact | (3): Very cost-effective (ICER=€5000 per life year gained); cost-effective (ICER=€50000 per life year gained); not cost-effective (ICER=€200000 per life year gained) |
| **van Dam, L., L. Hol, et al.** | **2010** | Colorectal cancer screening test | 1. Pain | (2): no pain; mild pain |
|  |  |  | 2. Risk of complications | (2): none; small |
|  |  |  | 3. Location of the screening test | (2): at home; hospital |
|  |  |  | 4. Preparation of the procedure | (4): none; enema; drinking of 0,75l of fluid and 12h fasting; drinking of 4l of fluid and 18h fasting |
|  |  |  | 5. Duration of the procedure | (4): 10min; 30min; 60min; 90min |
|  |  |  | 6. Screening interval | (4): 1xin 10 years; 2x in 10 years; 5x in 10 years; 10x in 10 years |
|  |  |  | 7. Risk reduction of CRC related death | (4): 2.7%(10% RR reduction); 1.8% (40% RR reduction); 1.2% (60% RR reduction); 0.3% (90% RR reduction) |
| **van Til, J. A., A. M. Stiggelbout, et al.** | **2009** | Ankle and foot impairment treatment | 1. Treatment duration | (4): 1 month; 3 months; 6 months; 9 months |
|  |  |  | 2. Treatment impact | (3): no surgery; surgery: implantation foreign materials; surgery:permanent changes in muscles |
|  |  |  | 3. Ease of use | (4): temporary aid; daily investment 3 min during treatment; temporary aid: daily investment 10 min durign treatment; permanent aid: daily investment 3 min; permanent aid: daily investment 10 min |
|  |  |  | 4. Complication type | (2): skin irritation; pressure scores |
|  |  |  | 5. Complication rate | (3): 1/100; 5/100; 10/100 |
|  |  |  | 6. Comfort and cosmetics | (4): invisible and imperceptible; perceptible, invisible; visible, imperceptible; visible and perceptible |
|  |  |  | 7. Result | (4): improved foot position with custom-made shoes; improved foot position and ankle stability with custom-made shoes; improved foot position with ready-made shoes; improved foot position and ankle stability with ready-made shoes, barefoot walking possible |
|  |  |  | 8. Success rate | (4):99/100;95/100;90/100;80/100 |
| **Waschbusch, D. A., C. E. Cunningham, et al.** | **2011** | Treatment for attention-deficit/hyperactivity disorder | 1. Parental skills | (3): does not help me manage my child more effectively; sometimes helps me manage my child more effectively; often helps me manage my child more effectively |
|  |  |  | 2. Parent's stress | (3): does not reduce my stress; sometimes reduces my stress; often reduces my stress |
|  |  |  | 3. Long-term side effects | (3): no long-term side effects; long-term side effects cause 15 of 100 parents to regret treatment;long-term side effects cause 30 of 100 parents to regret treatment |
|  |  |  | 4. Short-term side effects | (3): no short-term side effects; short-term side effects cause 15 of 100 parents to stop treatment;short-term side effects cause 30 of 100 parents to stop treatment |
|  |  |  | 5. Financial cost | (3): no cost; $3000/year; $6000/year |
